# Identification of a New Potent Inhibitor Targeting KRAS in Non-small Cell Lung Cancer Cells

**DOI:** 10.3389/fphar.2017.00823

**Published:** 2017-11-14

**Authors:** Chun Xie, Ying Li, Lan-Lan Li, Xing-Xing Fan, Yu-Wei Wang, Chun-Li Wei, Liang Liu, Elaine Lai-Han Leung, Xiao-Jun Yao

**Affiliations:** ^1^State Key Laboratory of Quality Research in Chinese Medicine, Macau Institute for Applied Research in Medicine and Health, Macau University of Science and Technology, Macau, China; ^2^State Key Laboratory of Applied Organic Chemistry and Department of Chemistry, Lanzhou University, Lanzhou, China

**Keywords:** KRAS, NSCLC, small molecule inhibitor, molecular docking

## Abstract

KRAS (v-Ki-ras2 Kirsten rat sarcoma viral oncogene homolog) is an oncogenic driver with mutations in 30% of non-small cell lung cancer (NSCLC). However, there is no effective clinical drug even though it has been identified as an oncogene for 30 years. In this study, we identified a small molecule inhibitor compound 0375-0604 targeting KRAS by using molecular docking based virtual screening approach. Compound 0375-0604 had a good binding affinity to KRAS *in vitro* and exhibited cytotoxicity in oncogenic KRAS expressing NSCLC cell lines. Further mechanism study showed that compound 0375-0604 can block the formation of the complex of guanosine triphosphate (GTP) and KRAS *in vitro*. In addition, compound 0375-0604 inhibited KRAS downstream signaling pathway RAF/MEK/ERK and RAF/PI3K/AKT. Finally, we also found that this compound can inhibit the cell growth through G2/M cell cycle arrest and induce apoptosis on the NSCLC cell lines harboring KRAS mutation. Therefore, compound 0375-0604 may be considered as a potential KRAS inhibitor for treatment of NSCLC carrying KRAS oncogene.

## Introduction

In lung cancer, NSCLC is the majority category and accounts for 85% (Ettinger et al., 2017). The overall survival of patients with advanced or metastatic NSCLC is still dismal (Shima et al., 2015; Lazo and Sharlow, 2016). With the development of modern sequencing technology, NSCLC was further classified into different subtypes according to the frequency of gene mutation, such as EGFR, ALK, MET, ROS-1, KRAS (Chen et al., 2014). Mutated KRAS genes are frequently found in human cancers, especially in approximately 30% of lung cancer. Ninety seven percent of mutated KRAS occurs in exon 2 and 3, including amino acid G12, G13, and Q61 (Karachaliou et al., 2013). Due to high morbidity and mortality, a great deal of attention has been paid to study NSCLC with KRAS mutations. However, there is still no direct and effective drug for clinical use (Jancik et al., 2010; Gysin et al., 2011; Vasan et al., 2014; Papke and Der, 2017).

KRAS plays an important role in normal cell development, such as proliferation and differentiation (Pylayeva-Gupta et al., 2011; Santarpia et al., 2012). As a small GTPase, KRAS normally cycles between inactive GDP-bound state and active GTP-bound state, which are tightly regulated by GTPase-activating proteins (GAPs) and Guanine nucleotide exchange factors (GEFs), respectively (Maurer et al., 2012; Burns et al., 2014; Leshchiner et al., 2015). However, mutant KRAS impairs its GAPs activity, which locks KRAS at the active state (Smith et al., 2013; Clausen et al., 2015). Thereby mutant KRAS promotes its interaction with a variety of effector proteins and activates downstream signaling events, and finally results in tumor formation (Bos et al., 2007; Zimmermann et al., 2013; Lito et al., 2016). Therefore, it is urgently needed to find effective inhibitors to target and inhibit oncogenic KRAS in cancers.

To date, there are mainly three strategies for the discovery of potent KRAS inhibitors: (1) to inhibit KRAS membrane targeting (Laheru et al., 2012; Prakash and Gorfe, 2013; Chavan et al., 2015; Cox et al., 2015); (2) to directly target KRAS (Wang et al., 2012; Cromm et al., 2015; Leshchiner et al., 2015; Brock et al., 2016; Trinh et al., 2016); (3) to inhibit interaction between KRAS and its downstream effectors (Athuluri-Divakar et al., 2016; Upadhyaya et al., 2016; Keeton et al., 2017). However, there are multiple escape pathways by process of post-translation for inhibiting KRAS membrane targeting (Rowinsky et al., 1999; Van Cutsem et al., 2004). Inhibitor lonafarnib and tipifarnib showed effective inhibition to KRAS mutations through blocking prenylation of RAS, but failed in clinical trial, as the geranylgeranylation could be in replacement of prenylation when the farnesyltransferase was inhibited by these two inhibitors (Berndt et al., 2011). Additionally, it may be not a good choice to inhibit interaction between KRAS and its downstream effectors for developing KRAS inhibitors. Firstly, there are a lot of downstream effector proteins of KRAS involving in multiple signaling pathways, such as RAF (MAP kinase pathway), PI3K (AKT/mTOR pathway), and RalGDS (Ral pathway). Secondly, these effectors are not only highly complex but also regulating multiple pathways (Downward, 2003). Arguably, designing a small molecule inhibitor directly targeting KRAS may be one of the most effective ways. However, the biggest challenges to develop direct KRAS inhibitor are the high binding affinity between KRAS and GDP/GTP in the picomolar range and the relatively flat surface without deep hydrophobic pockets in KRAS protein (Ledford, 2015; Vo et al., 2016). Notably, in recent years, several reported works have shown the novel transient pockets on KRAS protein surfaces, which recover the hope in the development of KRAS inhibitors (Prakash and Gorfe, 2013; Wang et al., 2014).

In this study, we aimed to identify effective and potential KRAS inhibitors by directly targeting KRAS to prevent cell growth of NSCLC harboring KRAS mutation. We performed a molecular docking-based virtual screening from a small molecule database to screen KRAS inhibitors. A potential inhibitor compound 0375-0604 was found to bind to KRAS and exhibit the effective cytotoxicity to KRAS mutant NSCLC cell lines.

## Results

### The Binding Mode between Compound 0375-0604 and KRAS

**FIGURE 1:**
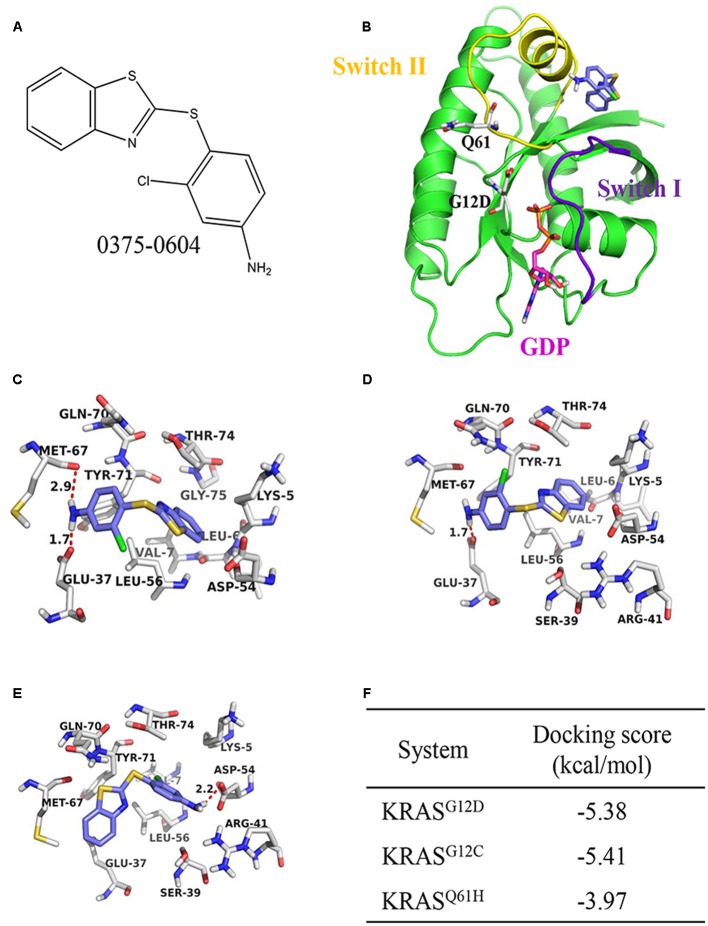
The structural analysis between KRAS protein and 0375-0604. **(A)** The chemical structure of 0375-0604. **(B)** Structure of 0375-0604 binds to GDP-KRAS^G12D^. The switch I and II region are colored in yellow and purple, respectively, while 0375-0604 and GDP are colored in blue and magenta, respectively. **(C–E)** Binding mode of 0375-0604 with KRAS^G12D^, KRAS^G12C^ and KRAS^Q61H^, respectively. **(F)** Docking score of 0375-0604 in various systems.

To discover potential small molecules targeting KRAS, virtual screening based on molecular docking was performed on Chemdiv library with about 1.36 million compounds. The most promising compound 0375-0604 (**Figure 1A**) was selected for further study. It was shown that the benzothiazole ring of 0375-0604 inserted into the binding pocket of KRAS with the linker sulfur atom exposed to solvent environment (**Figures 1B,C**). The amino group of compound 0375-0604 formed H-bond interactions with the backbone of Met67 and the side chain of Glu37, locating in switch I and II regions of KRAS^G12D^, respectively. At the same time, 0375-0604 formed polar contacts and hydrophobic contacts with the surrounding residues. 0375-0604 bound to KRAS^G12C^ with a similar manner (**Figure 1D**) as KRAS^G12D^, except for the orientation of chlorobenzene rings. The orientation of benzothiazole ring and chlorobenzene rings of 0375-0604 switched in KRAS^Q61H^ (**Figure 1E**). Docking score of 0375-0604 in various systems was shown in **Figure 1F**.

### The Binding Affinity of Compound 0375-0604 with KRAS

**FIGURE 2:**
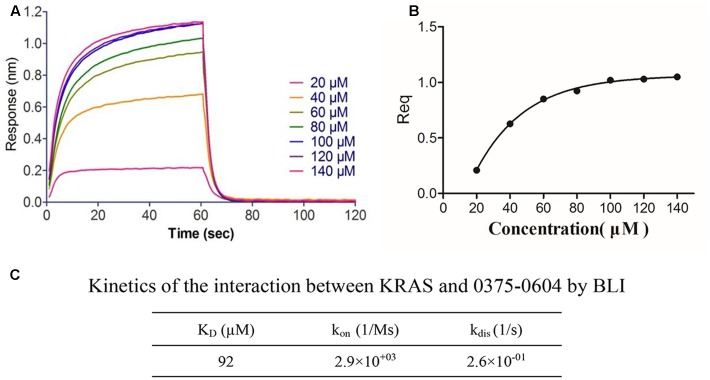
The binding affinity of compound 0375-0604 with KRAS was determined by interferometry studies. **(A)** Binding curves of varying concentrations of compound 0375-0604 to the immobilized KRAS protein. **(B)** Steady-state analysis of the binding curves. **(C)** The binding affinity (K_D_) of KRAS for compound 0375-0604 was determined. Experimental data for association and dissociation are represented as shown.

To determine the binding affinity of this small molecule with KRAS, we used biolayer interferometry assay (BLI) (Rich and Myszka, 2007), a label-free technology, to measure biomolecular interactions. Different concentration of compound 0375-0604 was measured in real time by association with both-labeled KRAS protein, which was immobilized on the streptavidin (SA) biosensors. All the association/dissociation binding curves was shown in **Figure 2A**, and we further performed the steady-state analysis (**Figure 2B**) with ForteìBio data analysis software to obtain the binding affinity with K_D_ value of 92 μM (**Figure 2C**), which demonstrated their direct and reversible interaction with KRAS.

### Compound 0375-0604 Decreased Cell Viability of NSCLC Cells with KRAS Mutations

**FIGURE 3:**
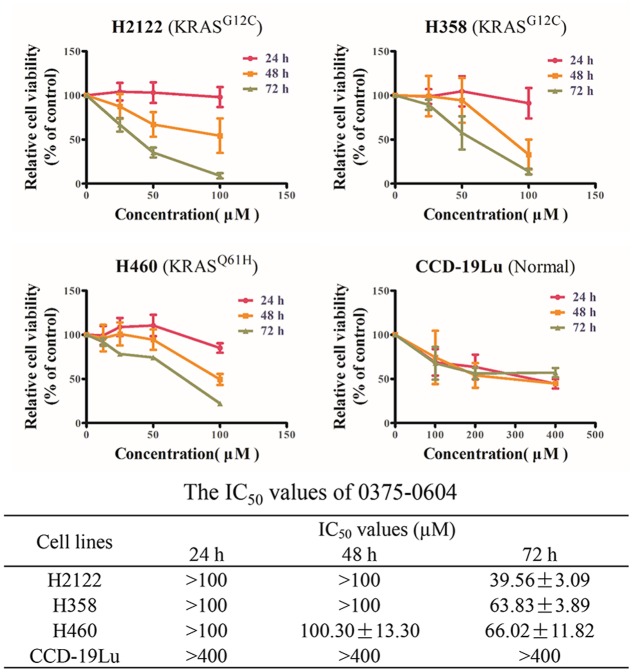
The cytotoxic effects of compound 0375-0604 on NSCLC cell lines were determined by MTT assay.

Since compound 0375-0604 bound to KRAS *in vitro*, we further determined its cytotoxicity in NSCLC cell lines harboring mutant KRAS by using MTT assay, including H2122 (KRAS^G12C^), H358 (KRAS^G12C^) and H460 (KRAS^Q61H^) cell lines. Cells were incubated with a range of compound 0375-0604 concentrations (0, 25, 50, 100 μM) for 24, 48, and 72 h. As shown in **Figure 3**, compound 0375-0604 inhibited three NSCLC cell lines in a dose- and time-dependent manner, but not in normal lung fibroblast cell line CCD-19Lu. Importantly, we found that the IC_50_ value of compound 0375-0604 in H2122, H358 and H460 cells were up to 6-fold less than that of CCD-19Lu cells, which suggested that compound 0375-0604 showed strong inhibition selectivity in NSCLC cells.

### Compound 0375-0604 Blocked GTP-KRAS Formation in NSCLC Cells

Actually, mutant KRAS would interfere the balance between GEFs and GAPs, resulting in locking in the active GTP-bound KRAS state and aberrant stimulation of its downstream signaling. Hence, KRAS inhibitors should reduce the formation of GTP-KRAS to disrupt the mutant KRAS function.

**FIGURE 4:**
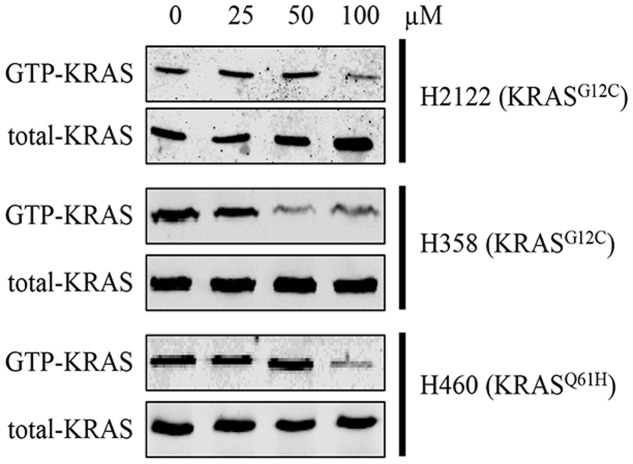
The inhibition of compound 0375-0604 on active KRAS. KRAS mutant cells (H2122, H358 and H460) were treated with compound 0375-0604 for 24 h. The level of active KRAS (GTP-KRAS) was determined by a RAS activation assay and immunoblotted with a KRAS-specific antibody.

In order to know whether compound 0375-0604 could inhibit activation of KRAS, we performed RAS activation assay to examine the formation of GTP-bound KRAS after treatment with a range of concentrations of compound 0375-0604 in H2122, H358 and H460 cells at 24 h. As shown in **Figure 4**, the formation of GTP-KRAS was inhibited in KRAS mutant NSCLC by compound 0375-0604 treatment, compared to total amount of KRAS, suggesting this small molecule could partially rescue this unbalance resulted from mutant KRAS.

### Compound 0375-0604 Inhibited the Activation of KRAS Downstream Signaling Pathway

**FIGURE 5:**
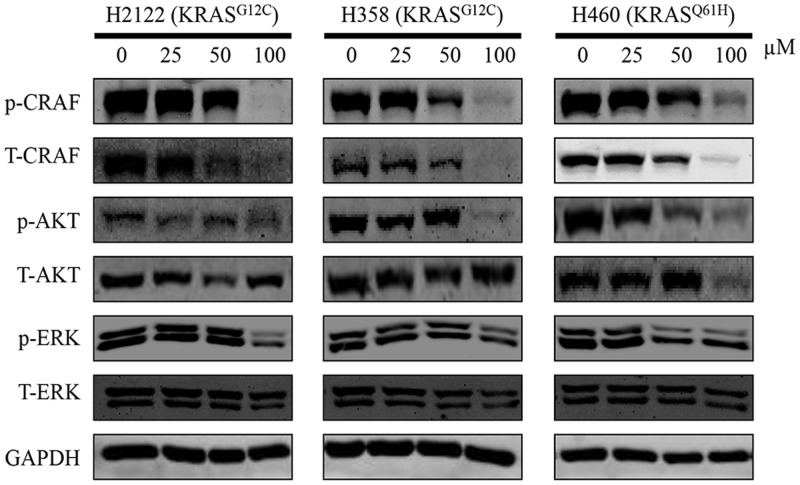
The inhibitive effect of compound 0375-0604 on the KRAS signaling pathways in different NSCLC cell lines. Three NCSLC cell lines were treated with compound 0375-0604 for 48 h. The levels of phospho(p)-CRAF, p-ERK, p-AKT, CRAF, AKT, ERK and GAPDH were determined by western blot analysis. Untreated cells were used as a control. A representative of at least three independent experiments for each cell line is showed.

Active KRAS stimulates downstream signaling pathways, especially for RAF/MEK/ERK and RAF/PI3K/AKT pathway, and then induces cell proliferation. Therefore, to investigate the effect of compound 0375-0604, we examined the phosphorylation levels of CRAF, AKT and ERK in NSCLC cell lines to monitor the impact of KRAS signaling by treatment with this compound for 48 h. As expected, compound 0375-0604 reduced the levels of phosphorylation of CRAF and AKT in a dose-dependent manner in all three NSCLC cell lines (**Figure 5**), which indicated that compound 0375-0604 may block oncogenic KRAS function through inhibiting its downstream signaling pathways.

### Compound 0375-0604 Induced Cell Cycle Arrest and Apoptosis in NSCLC

**FIGURE 6:**
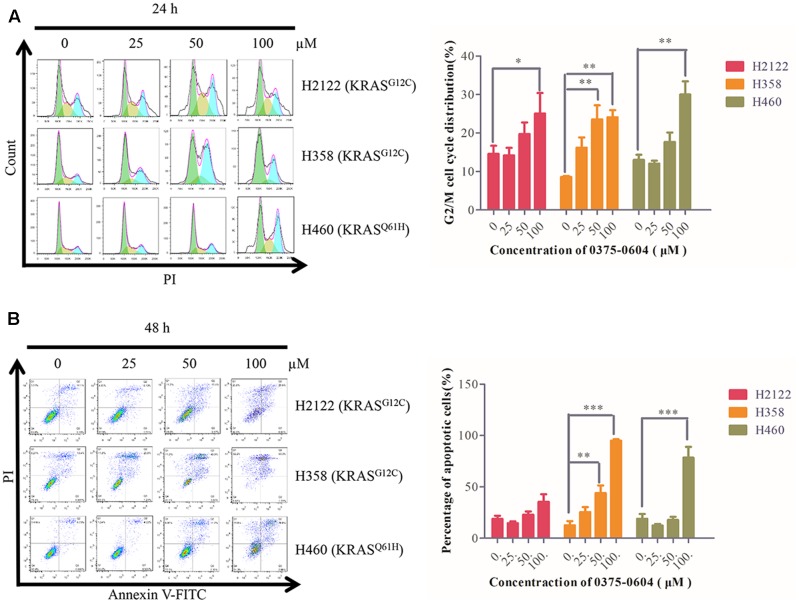
Cell cycle arrest and apoptosis were induced by compound 0375-0604 in three NSCLC cell lines (H2122, H358 and H460). **(A)** Flow cytometric analysis of cell cycle arrest with compound 0375-0604 at different concentrations (0, 25, 50, 100 μM) was examined for 24 h. **(B)** Flow cytometric analysis of cell apoptosis with different concentrations (0, 25, 50, 100 μM) of compound 0375-0604 for 48 h was determined.

Since compound 0375-0604 significantly inhibited cell viability of NSCLC cells with KRAS mutation, we examined whether compound 0375-0604 exhibited cytotoxicity by cell cycle arrest or apoptotic effect in H2122, H358 and H460 cells. Cells were treated with the indicated concentrations of compound 0375-0604 for 24, 48, and 72 h. Flow cytometric analysis showed that after 24 h treatment, the percentage of cells significantly decreased in G0/G1 phase while remarkably increased in G2/M phase (**Figure 6A**). In addition, compound 0375-0604 induced a significantly increased apoptosis for 48h in NSCLC cell lines (**Figure 6B**). These result suggested that compound 0375-0604 may block cell proliferation and cause cell death via induction of G2/M cell cycle arrest or/and apoptosis in H2122, H358, and H460 cells.

## Discussion

In this study, we identified and characterized a small-molecule compound 0375-0604 as a new KRAS inhibitor. By using molecular docking approach, we found that compound 0375-0604 bound to the switch regions (switch I and II) of KRAS. KRAS conformation changes and its downstream signals are activated when its switch regions, either switch I or switch II, interact with GTP. A remarkable feature of compound 0375-0604 is that it formed two hydrogen bonds interaction with the backbone of Met67 and the side chain of Glu37, which are located in switch I and switch II, respectively. These two key hydrogen bonds could partially stabilize the interaction of KRAS and 0375-0604. The docking calculation indicated compound 0375-0604 exhibited potent binding affinity with KRAS. In addition, the chemical structure of this inhibitor has more potential to be modified and achieve more potent and effective inhibition to oncogenic KRAS NSCLC. Based on the docking result, the binding affinity of compound 0375-0604 with KRAS protein was further determined by using BLI and K_D_ value was 92 μM, which suggested that 0375-0604 could bind to KRAS with good performance.

There are two most extensively RAS-mediated pathways: PI3K/AKT/mTOR and RAF/MEK/ERK pathway (Papke and Der, 2017). The PI3K/AKT/mTOR pathway represents an important intracellular signaling pathway, which involved in transition of cell cycle. It is directly related to cell proliferation, cancer progress and longevity (Bryant et al., 2014). The RAF/MEK/ERK pathway is a chain of proteins in cell that communicates a signal from a receptor on the surface of cell to the DNA in the nucleus of cell. The RAF serine/threonine kinases (ARAF, BRAF and CRAF) are arguably the most important effectors of mutant RAS-dependent cancer growth, as they have a key driver role in RAS-mediated oncogenesis. In our study, we found that compound 0375-0604 could reduce the activation levels of AKT, CRAF and ERK and block the activation of KRAS downstream signaling pathways in NSCLC.

KRAS binds to GTP in its active state and then influences the expression of downstream genes involved in crucial pathways on regulating cell growth, differentiation and apoptosis (Lu et al., 2016). Compound 0375-0604 showed a strong anti-cancer activity by inhibiting the activation of KRAS proteins, and caused G2/M cell cycle arrest at the early stage and induced apoptosis at the later stage in H2122, H358 and H460 cell lines harboring KRAS oncogene.

In summary, we identified a new small molecule compound 0375-0604 that bound to KRAS^G12D^, KRAS^G12C^ and KRAS^Q61H^ protein with a moderate binding affinity of -5.38, -5.41, and -3.97 kcal/mol, respectively. In addition, 0375-0604 selectively inhibited the proliferation of NSCLC cells with KRAS mutation but not normal lung cells. Compound 0375-0604 also blocked the formation of GTP-KRAS and downstream activation of KRAS *in vivo*. Besides, compound 0375-0604 inhibited the growth of cancer cells by causing G2/M cell cycle arrest and inducing apoptosis. Regardless, our study provides further evidence for targeting to KRAS protein, which may contribute to the future study for lung cancer therapy.

## Materials and Methods

### Molecular Docking

The KRAS^G12D^ structure (PDB code: 4DSU) complexed with GDP and a compound benzimidazole (BZIM) was used for the modeling of possible binding modes between KRAS and 0375-0604. The crystal structure with GDP was prepared in the Prep Wiz module of Maestro (Version 9.1, Schrodinger) and water molecules within 5 Å of het groups were kept. Subsequently, the residues of D12 and Q61 were mutated into C12 and H61 using the BioLuminate module of Maestro, respectively. A grid file was generated based on the position of compound BZIM in the Grid Generation wizard. Then, 0375-0604 was prepared to assign atomic charges and generate alternative conformations chemical rings. Finally, the docking process was employed in the Glide Docking module based on the previous obtained grid file using an extra precision (XP) protocol followed by a post-docking minimization using MM-GBSA method.

### Biotinylation

KRAS (Abcam, ab96817; 200 μg/ml) was biotinylated using the EZ-Link NHS-LC-LC-biotin (Thermo) in H_2_O using a 3:1 molar ratio of biotin reagent: protein for 30 min at room temperature following the FortéBio suggested protocol. Biotinylated KRAS was separated from the biotinylation reaction reagents by Zeba desalying spin columns (Thermo). Streptavidin biosensors (SA) tips (ForteìBio, Inc., Menlo Park, CA, United States) were prewetted with PBS to establish a baseline before immobilization.

### Biolayer Interferometry Analysis

A FortéBio Octet Red instrument was used in this assay. All the assays were performed at 96-well plate (Greiner Bio-One, PN:655209) and all the final volume for all the solutions was 200 μl/well. Biotinylated KRAS was immobilized onto SA tips. The experiments comprised three steps: (1) baseline, (2) association, (3) dissociation. The association and dissociation plot and kinetic constants were obtained with ForteìBio data analysis software. For measurement the interaction between compound 0375-0604 and KRAS, seven concentrations of compound 0375-0604 (20, 40, 60, 80, 100, 120, 140, 160 μM) were used for association step.

### 3-(4, 5-Dimethylthiazol-2-yl)-2, 5-Diphenyltetrazolium Bromide (MTT) Assay

H2122, H460 and H358 were purchased from ATCC and cultivated with RPMI 1640 medium supplemented with 10% fetal bovine serum (FBS), 100 units/mL penicillin and 100 μg/mL streptomycin. All the cells were cultivated at 37°C with 5% CO_2_ incubator. Cells were seeded on a 96-well microplate with 3000, 4000, or 5000 cells/well, and were cultured overnight for cell adhesion. After add a range of compound 0375-0604 the microplates put back into incubator and incubated for 24, 48, or 72 h. Each dosage was repeated as triplicate. 10 μL MTT (5 mg/mL) solutions were added to each well. After incubated 4 h 100 μL DMSO was added to each well. After 15 min shake absorbance of the plate was measured at 570 nm (absorbance) and 650 nm (reference) by a microplate reader (Tecan).

### Pull Down Assay

RAS activity was determined using RAS activation assay kit (EMD Millipore, 17–218). Briefly, lysates were incubated with glutathione S-transferase fusion of the Ras binding domain (RBD) of Raf1 along with glutathione agarose for 1 h. Agarose beads were collected by centrifugation and washed three times with Mg^2+^ lysis buffer. Each sample were resuspended and boiled for 5 min. Finally, samples were subjected to western blotting as previously described and blots probed using anti-KRAS antibody (Santa, sc-30).

### Western Blot Analysis

After 48 h treatment with compound 0375-0604, RIPA lysis buffer (150 mM NaCl, 50 mM Tris pH 7.4, 1 mM EDTA, 0.25% sodium deoxycholate, 1% NP-40) containing protease (Roche) and phosphatase (Roche) inhibitors was added to extract the total whole cell protein. Bio-Rad DC^TM^ protein assay kit was used to quantify the concentration of extract protein. Thirty microgram protein lysate was loaded and separated by 10% SDS-polyacrylamide gel electrophoresis and transferred to a nitrocellulose (Millipore) membrane. The membrane was incubated with the primary antibody (1:2000) and then with a fluorescence-conjugated secondary antibody (1:10000). GAPDH was used as the loading control and for normalization. The signal of the membranes was scanned with a LI-COR Odyssey Scanner (Belfast).

### Cell Cycle and Apoptosis Assay Using Flow Cytometry

H2122, H358 and H460 cells were plated on a 6-well plate with 1.5 × 10^5^ cells/well and cultured overnight for attachment. After treatment with compound 0375-0604 at 0, 25, 50, 100 μM for 24, 48, and 72 h, all cells were harvested and collected. For cell cycle analysis, cells pellets were re-suspended in 70% ethanol and fixation at 4°C for 30 min. Each cell pellet was stained in 300 μL propodium iodide (PI) (50 μg/ml) staining solution at 37°C for 30 min in dark. Then, cells were washed twice with PBS. Finally, cells were re-suspended in 300 μL PBS and transferred to the flow cytometer (FACSCalibur, BD Biosciences). For apoptosis analysis, cells will re-suspended with 100 μL annexin-binding buffer, stained with annexin V and PI staining solution and incubated 15 min at room temperature protect from light. Finally, cells were diluted in 300 μL annexin-binding buffer and quantitatively measured using flow cytometer (FACSCalibur, BD Biosciences).

### Statistical Analysis

Descriptive analytical data are presented as means ± SD. Statistical analysis was conducted using Graph Prim 5.0. Significant differences between datasets were assessed by one-way analysis of variance (ANOVA).

## Author Contributions

X-JY, EL-HL, and LL conceived the project. X-JY, EL-HL, and CX designed the experiments. CX, YL, L-LL, X-XF, Y-WW, and C-LW carried out the research and analysis of data. X-JY, EL-HL, LL, and CX wrote the paper.

## Conflict of Interest Statement

The authors declare that the research was conducted in the absence of any commercial or financial relationships that could be construed as a potential conflict of interest.
